# Isotope Ratio Outlier Analysis (IROA) for HPLC–TOFMS-Based Metabolomics of Human Urine

**DOI:** 10.3390/metabo12080741

**Published:** 2022-08-12

**Authors:** Fadi Fadil, Claudia Samol, Raffaela S. Berger, Fabian Kellermeier, Wolfram Gronwald, Peter J. Oefner, Katja Dettmer

**Affiliations:** Institute of Functional Genomics, University of Regensburg, 93053 Regensburg, Germany

**Keywords:** metabolomics, metabolic fingerprinting, stable isotope, HPLC, mass spectrometry, labeled yeast extract, IROA, relative quantification

## Abstract

Metabolic fingerprinting by mass spectrometry aims at the comprehensive, semiquantitative analysis of metabolites. Isotope dilution, if successfully implemented, may provide a more reliable, relative quantification. Therefore, the ^13^C labeled yeast extract of the IROA TruQuant kit was added as an internal standard (IS) to human urine samples measured in full-scan mode on a high-performance liquid chromatography-time-of-flight mass spectrometer (HPLC–TOFMS) system. The isotope ratio approach enabled the analysis of 112 metabolites. The correlation with reference data did not improve significantly using ^12^C/^13^C ratios compared to absolute ^12^C peak areas. Moreover, using an intricate ^13^C-labeled standard increased the complexity of the mass spectra, which made correct signal annotation more challenging. On the positive side, the ratio approach helps to reduce batch effects, but it does not perform better than computational methods such as the “removebatcheffect” function in the R package Limma.

## 1. Introduction

Metabolic fingerprinting, also called untargeted metabolomics, aims at analyzing all detectable metabolites in a given sample. It is a semiquantitative approach that focuses on spotting significant differences in metabolite abundance among different groups of a sample set [1,2,3,4,5]. To this end, liquid chromatography–mass spectrometry (LC–MS) is widely used.

Issues such as matrix effects (i.e., the variability of ionization efficiency); analytical variability, e.g., decline in instrument performance when analyzing large sample batches; and difficulties in annotating the detected features are hurdles of this approach [6,7,8]. Metabolic profiling, on the other hand, circumvents these obstacles by using known amounts of labeled internal standards (IS), and tailored sample preparation and LC–MS methods for the optimal detection and absolute quantification of a predefined but often relatively small set of target metabolites [9,10]. Often relative, as in metabolic fingerprinting, instead of absolute quantification is sufficient. In this context, stable isotope-labeled internal standards can improve analytical repeatability and allow for the relative quantification of a large number of metabolites in a single LC–MS run. The question remains, which internal standards are best to use when all the detectable metabolites are targeted? One approach is to use a stable isotope-labeled reagent to derivatize a sample aliquot or a pool sample to generate a sample specific IS [11,12,13,14]. Yet, this approach is only selective to certain compound classes that are covered by the chosen derivatization. Global labeling of the whole metabolome of an organism was used in targeted [15] and untargeted [16] analyses, but it was limited to the respective organism. Dethloff et al. [17] used partially labeled mouse plasma as an IS for amino acid analysis in human plasma. Furthermore, ^13^C-labeled yeast extracts have been used (ISOtopic solutions, Vienna, Austria) [9]. Employing a complex ^13^C labeled yeast extract is also the basis of the IROA TruQuant kit (IROA Technologies LLC^®^, Bolton, MA, USA).

Isotopic ratio outlier analysis (IROA) is a mass-spectrometry-based technique that helps to distinguish features of biological origin from nonbiological artefacts and, secondly, aims at providing more reliable quantitative data by correcting for ion suppression and analytical variance [18]. The kit includes two types of standards. The long-term reference sample (LTRS) is a fully labeled *S. cerevisiae* yeast cell extract, labeled at 5% and 95% ^13^C and mixed 1:1. This translates to a unique isotopic pattern for all features of a biological origin, whereas artefacts would exhibit the isotopic pattern of natural abundance, allowing for a distinct filter criterion. Note that the LTRS is injected *as is*, without mixing with any specimens of interest. Thereby, the LTRS serves as a quality control (QC) for instrumental performance. It also allows for a validated annotation of the detected features that show the expected isotopic pattern, yielding a reference library. The second standard is the internal standard (IS), which only contains the 95% ^13^C labeled yeast extract. It is added to the samples to be analyzed, providing the advantages of a traditional internal standard by correcting for ion suppression and analytical variance. It also offers a more robust annotation process, benefiting from the reference library built using the LTRS measurements [6,18]. The key advantage compared to a traditional internal standard is that it comprises a very large number of labeled compounds, i.e., all metabolites present in yeast. However, as the exact quantities of the labeled compounds are not known, it allows for relative but not for absolute quantification.

Metabolomics data acquired in separate experimental batches may exhibit variation unrelated to biological differences among different sample groups. The sources of this additional analytical variance are, for example, varying experiment times, reagents, and instrument performance. Even when using the same reagents and instruments, the performance of the high-performance liquid chromatography (HPLC) system and the mass spectrometer response may deviate over time. These effects are aptly termed ‘batch effects’ [19]. Their impact can be mitigated using batch-effect correction algorithms (BECAs) such as “removebatcheffect” (RBE), which is a function in the Limma package in R [20,21], where the batch effect is modeled by an additional coefficient. However, they can only be applied in cases where data have been acquired in distinct batches and not in cases where samples were measured continuously over prolonged periods of time. Furthermore, BECA’s effectiveness is questionable in some cases as they could be misapplied, and the fitted model might introduce bias into the data [19]. Taking measures early in the workflow might produce better results than circumventing batch effects via meta-analysis. Having a reference sample such as the LTRS could help to account for changes in chromatographic conditions, instead of using more lenient thresholds in feature alignment and weak signal recovery [22]. The implemented IS can also correct for shifts in the detector response.

Several studies [6,23,24] have employed the isotopic ratio outlier analysis technique in model organisms. In such experiments, only one type of sample matrix is involved, and any features that do not exhibit the IROA isotopic pattern are indeed artefactual. Here, however, we used the labeled yeast extract, i.e., the IS of the TruQuant kit, as an internal standard to analyze human urine specimens. Thus, we are dealing with two different matrices. If a feature does not exhibit the IROA isotopic pattern, it could merely be a compound that is urine-specific or not produced in yeast in detectable quantities. With this in mind, we investigated the added value of incorporating the IROA TruQuant kit in our untargeted analysis of human urine.

We analyzed a set of 244 spot urine specimens from the German Chronic Kidney Disease (GCKD) project collected at the baseline time point [25]. The samples were analyzed by means of a high-performance liquid chromatography (HPLC) system coupled to a time-of-flight mass spectrometer (TOFMS). The resulting data are referred to as TOF. To generate reference datasets, a subset of 56 of the urine specimens was additionally analyzed with two different methods. The first was MS-based quantitative data of amino acids (AAs), referred to as “quant”. The second, referred to as “NMR”, stemmed from a nuclear magnetic resonance (NMR) quantitative analysis.

## 2. Materials and Methods

### 2.1. Chemicals

The IROA TruQuant IQQ Workflow Kit was purchased from Sigma–Aldrich (Taufkirchen, Germany). HPLC grade solvents for LC–MS analysis were purchased from VWR (Vienna, Austria). Purified water used to prepare the solutions was from a PURELAB Plus system (ELGA, LabWater, Celle, Germany)

### 2.2. Sample Preparation

The IROA Kit was applied to 244 urine samples from the German Chronic Kidney Disease (GCKD) project [25], from the baseline visit. The study was executed in accordance with the Declaration of Helsinki and registered in the national registry for clinical studies (DRKS 00003971). All study procedures and protocols were approved by the ethics committees of all participating institutions (Friedrich Alexander University Erlangen–Nuremberg, the Medical Faculty of the Rheinisch–Westfälische Technische Hochschule Aachen, Charité University Medicine Berlin, the Medical Center University of Freiburg, Medizinische Hochschule Hannover, the Medical Faculty of the University of Heidelberg, Friedrich Schiller University Jena, the Medical Faculty of the Ludwig Maximilians University Munich, and the Medical Faculty of the University of Würzburg). The study was carried out in accordance with relevant guidelines and regulations. Written declarations of informed consent were obtained from all study participants before inclusion. All specimens were stored at −80 °C until preparation of samples for mass spectrometry.

Various factors affect the abundance of urinary metabolites, such as liquid and food intake and many processes regulating the body’s solute and water content. Normalization is needed to balance out these differences among individuals and reveal actual biological variance. Creatinine is commonly used to achieve that as it is mainly excreted by glomerular filtration at a constant rate without reabsorption in the renal tubule [26]. Urine was diluted with pure water to a similar creatinine concentration to correct for urinary output. Since the individual dilution of each sample is impractical, samples were grouped based on the creatinine concentration, and a fixed dilution factor was used for the respective group, see Table 1. Creatinine concentrations determined in the course of a corresponding NMR analysis of the samples were utilized.

Samples were diluted to a volume of 20 µL. For the internal standard, five vials of IROA–IS were used. To each vial, 600 µL of pure water was added, instead of the recommended 1200 µL, to keep a proper concentration after dilution with urine. All five resulting IS solutions were mixed to form a homogenous 3 mL IROA–IS solution. Then, 10 µL of IROA–IS was added to each diluted urine sample, resulting in a final volume of 30 µL. Two in-house QC urine samples were prepared similarly. QC1 and QC2 have creatinine concentrations of 19.71 mM and 8.92 mM, respectively, and were diluted 1:8 and 1:4 with water correspondingly, to reach a volume of 120 µL. Sixty microliters of IS was then added to each QC, which allowed one to maintain the same sample:IS ratio used to prepare the samples. The final volume of 180 µL of each QC was necessary to allow multiple injections throughout the experiment.

For the LTRS, 80 µL of water was added to reach a similar concentration of the IS in the samples. IS blank was prepared by adding 80 µL water to a 40 µL IS solution. A water blank was included.

For NMR analysis, samples were prepared by mixing 400 μL human urine with 200 μL of 0.1 M phosphate buffer, pH 7.4, which contained 3.9 mM boric acid to impair bacterial growth. Furthermore, 50 μL of 0.75% (wt) trimethylsilylpropanoic acid (TSP) in deuterium oxide (D_2_O) and 10 μL of 81.97 mM formic acid (FA) were added as internal reference standards. NMR spectra were acquired on a Bruker 600 MHz Avance III HD NMR spectrometer (Bruker BioSpin GmbH, Rheinstetten, Germany) equipped with a helium cooled cryo–probe and an automated sample changer. For each sample, a 1D ^1^H spectrum was acquired employing a Carr–Purcell_Meiboom–Gill (CPMG) pulse sequence to facilitate the suppression of macromolecular signals. From the obtained spectra, metabolites were quantified by the fitting of reference signals employing the Chenomx NMR suite v. 8.6 (Chenomx Inc., Edmonton, Canada).

### 2.3. Instrumentation

A Thermo Scientific Dionex Ultimate 3000 UHPLC system (Idstein, Germany) was coupled through an ESI source to a Maxis Impact QTOFMS (Bruker Daltonics, Bremen, Germany). Chromatographic separation was accomplished with a Waters™ Atlantis™ PREMIER BEH C18 AX (1.7 µm 150 × 2.1 mm id) column kept at 35 °C. The aqueous eluent A is formic acid 0.1% (*v*/*v*) and 1 mM ammonium formate in water. The organic eluent B is formic acid 0.1% (*v*/*v*) and 10 mM ammonium formate in water: ACN (10:90, *v*/*v*). A flow rate of 0.3 mL/min was used. Elution was accomplished by the following eluent B gradient: 0–30% in 10 min, 30–100% in 5 min, 100% for 5 min, back to 0% in 2 min, followed by equilibration (0% B) for 8 min. Sample volumes of 5 µL were injected.

Electrospray ionization was performed in positive ionization mode using the following settings to operate the source and the mass spectrometer: a drying gas (nitrogen) temperature of 220 °C and a flow rate of 10 L/min; nebulizer gas pressure (nitrogen): 2.6 bar; end plate offset: 500 V; capillary voltage: 4500 V; mass range: 50–1000 *m*/*z*; and acquisition rate: 2 spectra/s. An external calibration of the mass spectrometer was employed before the measurements, using a sodium formate cluster solution (10 mM sodium formate in 50:50 *v*/*v* water/isopropanol). For internal recalibration, each run involved an injection of the sodium formate cluster solution through a six-port valve. A mass spectral resolution of R = 21,000 (for *m*/*z* 90.977) was obtained. The samples were injected in random order. On average, QCs were injected after every 20 samples, whereas LTRS, IS blank, and water blank were injected after every 40 samples. In total, 331 injections were performed.

### 2.4. Analysis of Amino Acids

Absolute concentrations of amino acids were determined by HPLC–MS/MS, using Agilent 1200 series HPLC system (Agilent, Waldbronn, Germany) with API 4000 QTRAP (AB SCIEX, Darmstadt, Germany) after derivatization with propyl chloroformate/1–propanol using 10 μL urine. Deviating from the established protocol [27], the derivatizing reagent 2 was composed of 17.4% propyl chloroformate and 82.6% isooctane, replacing chloroform entirely with isooctane.

### 2.5. Software

DataAnalysis version 4.1 (Bruker Daltonics, Bremen, Germany) was used for manual inspection and the processing of the HPLC–TOFMS mass spectra and chromatograms, internal recalibration of the spectra, and picking accurate masses. MSConvert Version: 3.0.19 (a tool of ProteoWizard; Palo Alto, CA, USA) [28] was used to convert the files to .mzxml format, using the parameter “vendor” of the peak picking filter. The IROA kit comes with dedicated software, ClusterFinder™ V3 (IROA Technologies LLC^®^, Bolton, MA, USA). It was used to establish a library based on the measurements of the LTRS runs. The untargeted analysis was done using MZmine 2 [29], where the chromatographic peaks were detected, automatically integrated, deconvoluted, aligned, and filtered. The integrated peak areas were extracted for further statistical evaluation.

### 2.6. Data Processing

Internal recalibration using the sodium formate cluster allowed for a mass tolerance of only 3 mDa. The LTRS data files were evaluated in ClusterFinder, and a reference library was created. This library consists of the peak pairs of the main ^12^C and ^13^C peaks, i.e., the unlabeled and the fully labeled peaks of each compound, respectively. ClusterFinder did not manage to handle all 331 data files at once. Therefore, further data evaluation had to be performed in MZmine 2 using the reference library to identify peak pairs. Peak pairs were matched manually, and orphan entries were excluded. Further data curation was done based on quality control. For instance, the ^12^C peaks should be minimal in the IS blank, so if a considerable ^12^C peak (^12^C/^13^C > 20%) was detected there, the pair was excluded. Additionally, the ratio of the pairs in the LTRS samples should be very close to one; if it deviated by more than 30%, the pair was excluded too. To confirm the annotations of the metabolites, a cross-identification was performed using our in-house library, generated using the Mass Spectrometry Metabolite Library (MSMLS™) (IROA Technologies LLC^®^, Bolton, MA, USA).

### 2.7. Data Evaluation

To assess the added value of using the kit, the data was evaluated using either the absolute peak areas of the ^12^C signal, referred to as “TOF absolute”, or the ^12^C/^13^C ratios, referred to as “TOF ratios”. ^12^C peak areas were corrected for the dilution factor. The data were compared to data from two other sources. Quantitative data (targeted analysis) acquired for the same sample set, measured in-house on the 4000 QTrap in MRM mode, referred to as “quant”. The other data set for the same sample set was also produced via an in-house NMR analysis, referred to as “NMR”.

**Figure 1:**
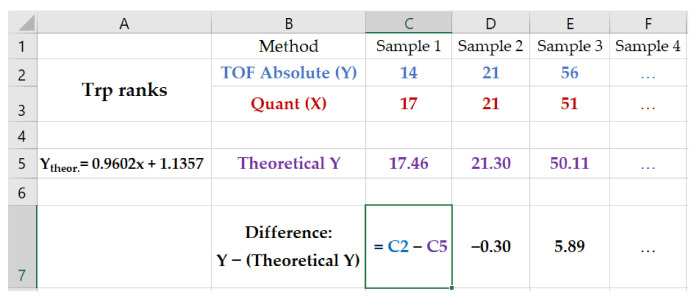
Computation of “diff” values. An exemplary Spearman correlation for Tryptophan (Trp) is calculated between TOF absolute and quant. The equation of the fitted regression line is shown on the left in field 5A. Note that this equation corresponds to the equations of regression given in Figure 2. From the assigned TOF absolute ranks (in blue), the theoretical ranks (i.e., the points on the regression line shown in purple) are subtracted, yielding the row “Difference”, which indicates the individual deviations from the regression line.

The IS would have an added value if the TOF ratios data correlated better to the other two datasets than the TOF absolute values. Spearman correlation coefficients were calculated. Spearman correlations were plotted, and a regression line was fitted. To analyze whether there are significant differences in the agreement of the TOF absolute or the TOF ratio method with either the quant or NMR method, we computed for both cases in each data set individual differences (“diff” values) from the respective regression lines (see Figure 1 for a typical example). These sets of differences were then analyzed by means of statistical tests. First, a Shapiro–Wilk normality test was performed to see if values in each set were normally distributed. If they were, a paired t-test was performed; otherwise, a paired Wilcoxon test was performed.

## 3. Results

Across all samples, 423 potential IROA features were found out of 12,938 unknown features. Quality control based on accurate masses, retention time, and feature intensity (see Section 2.6 Data processing) was applied to filter out and match the IROA ^12^C and fully labeled ^13^C signals. This left 224 features, resulting in 112 IROA pairs. Using our in-house retention time library, the identity of 27 out of these 112 were confirmed, see Table S1.

**Figure 2:**
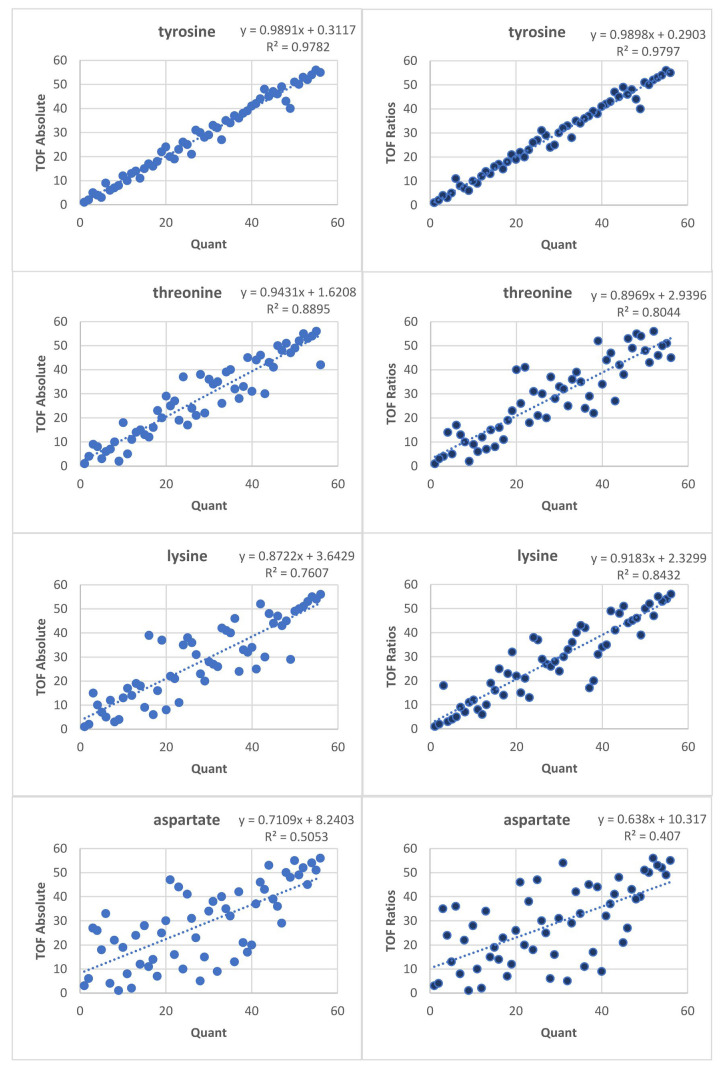
Spearman correlation plots of TOF absolute vs. quant (**left**), and TOF ratios vs. quant (**right**) (ranks are shown). The four amino acids (AAs) range from showing high correlation (**top**) to low correlation (**bottom**).

**Figure 3:**
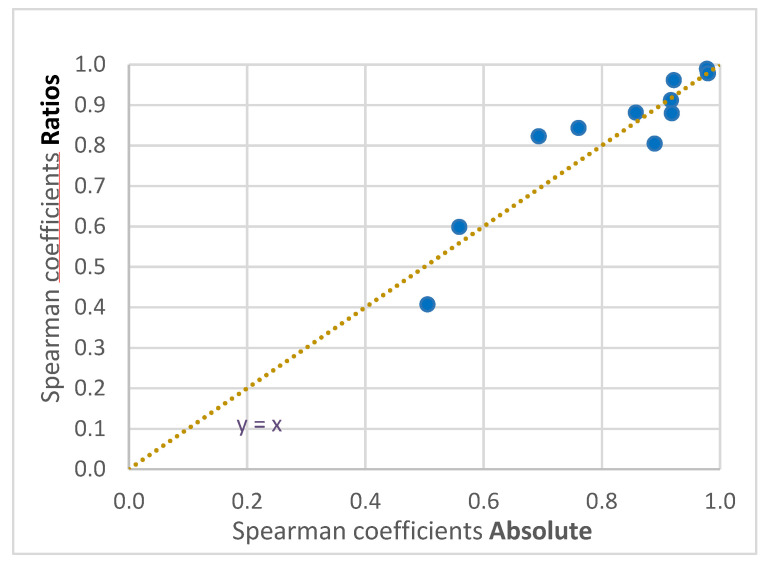
Scatter plot of Spearman correlation coefficients for TOF absolute vs. quant (*x*-axis) and TOF ratios vs. quant (*y*-axis) of the mutual metabolites between quant and TOF (see Table 2). Note that the dotted line represents the diagonal and not a regression line.

To assess the added value of using the kit, the data were evaluated once using the absolute peak areas of the ^12^C signal, referred to as “TOF absolute”, and then again using the ratios to the respective internal standards (^12^C/^13^C), referred to as “TOF ratios”. Subsequently, AAs detected with the two other methods, ‘‘quant’’ and “NMR”, see Table S1, were compared to both “TOF absolute” and “TOF ratios”. Leucine and isoleucine were chromatographically improperly resolved in the TOF data; hence, they were excluded, leaving 11 AAs covered both by quant and TOF absolute/TOF ratios. Figure 2 shows the Spearman correlation plots of four exemplary AAs ranging from high correlation (top) to low correlation (bottom), where the TOF data is represented by either TOF absolute (left) or TOF ratios (right) and compared to quant. Similar comparisons against quant were performed for all 11 mutual AAs. The corresponding Spearman correlation coefficients are given in Table 2. Neither substantial differences using TOF absolute versus TOF ratios nor a clear trend can be seen. For a better visual comparison, the coefficients of both comparisons for all 11 AAs were plotted in a scatter plot, see Figure 3.

To analyze whether there are significant differences in the agreement of the TOF absolute or the TOF ratio method with either the quant or NMR method, we computed, for both cases in each data, set individual differences (“diff” values) from the respective regression lines (see Section 2.7 Data evaluation). These sets of diff values were then analyzed by means of statistical tests. In case of normally distributed diff values (Table S2) a *t*-test was used; otherwise, a paired Wilcoxon test [30] was performed. The results shown in Table 3 suggest no significant difference between the correlation of TOF absolute and TOF ratios against quant.

**Figure 4:**
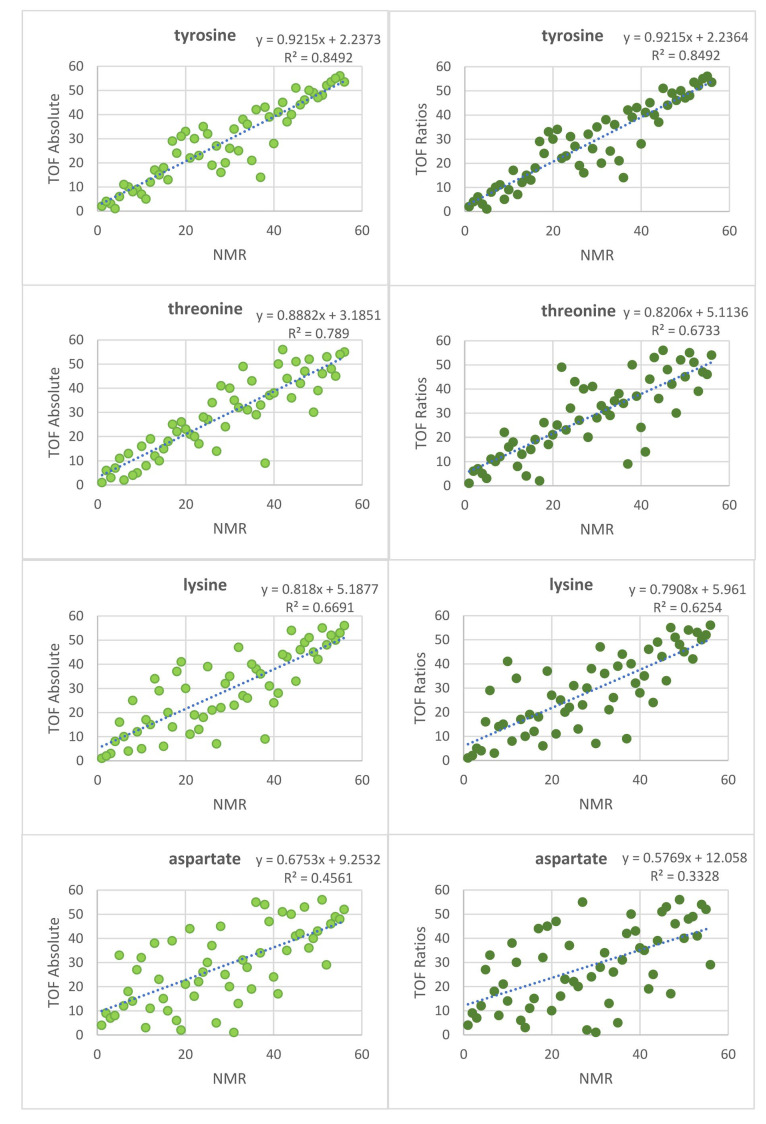
Spearman correlation plots of TOF absolute vs. NMR (**left**), and TOF ratios vs. NMR (**right**) (ranks are shown). The four AAs range from showing high correlation (**top**) to low correlation (**bottom**).

Similar comparisons were performed for six AAs that could be quantified by NMR (Table S1). Figure 4 shows the Spearman correlation plots for the comparisons of TOF absolute and TOF ratios to NMR for four exemplary AAs. The Spearman correlation coefficients of the six AAs are shown in Table 4. The “diff” values were also calculated for each dataset. The results of paired Wilcoxon or *t*-tests are shown in Table 5. Similar to quant, no significant difference between “TOF absolute” and “TOF ratios” in the correlation is seen. Furthermore, Figure S1 shows Spearman correlation plots between “NMR” and “quant” for the six overlapping AAs.

Overall, a poor correlation was observed for aspartate and proline in all comparisons due to the low aspartate and proline concentrations in urine, see Figure S2. The correlation with the NMR data was overall lower, which can be attributed to difficulties in NMR data analysis due to overlapping signals.

To further investigate the differences between TOF absolute and TOF ratios, relative standard deviation (RSD) values of the QCs were considered in both datasets. Note that two urine samples served here as QCs and were analyzed repeatedly throughout the batch, resulting in 14 measurements each. The RSDs of the 112 features were calculated as TOF absolute and TOF ratios for QC1 and QC2 separately; then, in addition, the average RSD of both was calculated, resulting in a total of six lists of RSD values. Figure S3 shows histograms of the average RSD values for TOF absolute and TOF ratios. TOF absolute exhibits more features in the lower RSD range. Statistical analysis was performed to check whether the difference is significant. Shapiro–Wilk normality tests [31] for all six lists of RSD values show significant *p*-values (Table S3). Thus, paired Wilcoxon tests were conducted (Table S4). The resulting *p*-values together with the data presented in Figure S3 suggest that the RSD in TOF absolute is significantly lower than in TOF ratios. This can be caused by the erroneous automatic peak integration of the internal standard peaks, which will in turn lead to erroneous ratios. In general, abundant analytes with peaks way above noise level are less challenging for automatic peak integration algorithms, and vice versa. To further investigate that, the 112 features were divided into four quantiles (Q1–Q4) based on the peak area of the respective internal standards. Q1 contains the features with the largest IS peak areas, and Q4 has the lowest. Shapiro and paired Wilcoxon tests between the average RSD values in each quantile were conducted, see Table S5. Q1 shows a nonsignificant Wilcoxon *p*-value, suggesting that features with the abundant IS signal show no significant difference between absolute and ratios. Although Q2 already shows a significant *p*-value, it is higher than those of Q3 and Q4. Individual features were checked, and the three features with the highest RSD in TOF ratios were inspected. The first one shows integration errors despite having an abundant IS; this is justified by the isomer coeluting close to the IS peak, Figure S4. The second has a mismatch between the endogenous feature and its IS. The third has again a closely eluting compound interfering with the integration.

The ratio approach was also tested for its usability for batch-effect correction. For this, a subset of 56 samples randomly drawn from the original set of 244 measurements were denoted Batch I. The corresponding 56 samples were measured again, six months later, using the same methods (Batch 2). As a comparison, the batch effect–correction algorithm “removebatcheffect” (RBE) of the Limma package was tested [21].

**Figure 5:**
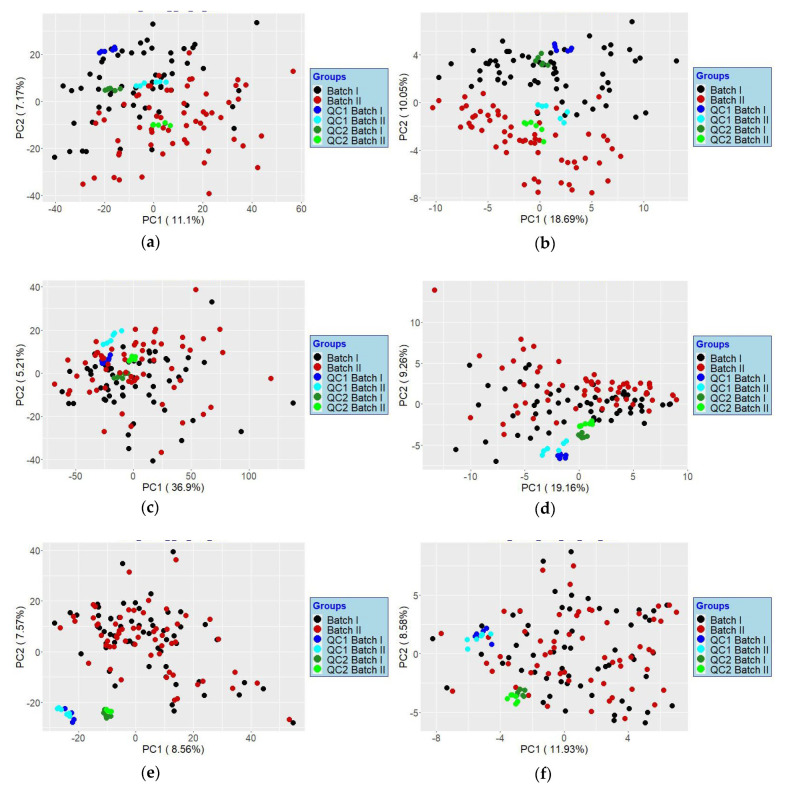
PCA score plots based on the (**a**) T-list, (**b**) IROA–Abs list; (**c**) T-list after applying PQN and Z-transformation; (**d**) IROA–ratio list; (**e**) T-list after applying PQN and RBE; (**f**) IROA–Abs list after applying PQN and RBE. For the same QC, the closer the injections coming from both batches cluster together, the more indicative it is that batch effects have been mitigated.

Two feature lists were created for the comparison containing the data from both batches each. The first is the feature list yielded by the typical fingerprinting analysis workflow, containing all detectable features meeting the analysis parameters. It comprised 3291 unidentified features and is referred to as the “T–list”. To account for RT shifts between the two batches, a higher than usual RT tolerance (0.3 min instead of 0.2 min) was used for peak alignment in the T-List. The second list was a feature list of metabolites that were identified using the IROA kit. It comprised 115 metabolites showing the IROA isotopic pattern, which were detected in both batches, and their respective internal standards. The LTRS measurement in both batches was used to identify and align the metabolites. From the second list, two further lists were generated. The list comprising the absolute peak areas of the metabolite without considering their ISs is referred to as the IROA–Abs list. The list in which the peak areas of the metabolites were divided by those of their corresponding ISs is called IROA–ratio list. The PCA score plots of T–list and IROA–Abs list showed a pronounced shift between the two batches (Figure 5a,b). This can be clearly observed by considering the clustering of the respective QC samples. The closer the clusters of the same QC, the less pronounced are the batch effects, and vice versa. Leek et al. demonstrated that normalization alone does often not remove batch effects [32]. We applied the probabilistic quotient normalization (PQN) [33] followed by a Z–transformation for each batch separately in the T-list. This reduced the shift in the QC samples (Figure 5c). Using the IROA–ratio list also provided a considerable improvement over the IROA–Abs list, Figure 5d. However, applying PQN followed by RBE produced the best results, whether on the T-list or the IROA–Abs list, with data points from the two batches pairing well and QCs clustering more closely (Figure 5e,f).

## 4. Discussion

The data shows no significant improvement if TOF ratios are used instead of the TOF absolute when comparing the results to reference measurements. This poses the pivotal question of whether the use of an IS in untargeted LC–MS metabolomics carries any benefit. The answer lies within the nature of the metabolic fingerprinting approach itself. Trying to be as universal as possible makes one miss out on the perks of tailored methods that are designed to take full advantage of having an IS. This includes manual peak integration, or the manual inspection of automatically integrated peaks, which cannot be practically applied to metabolic fingerprinting.

**Figure 6:**
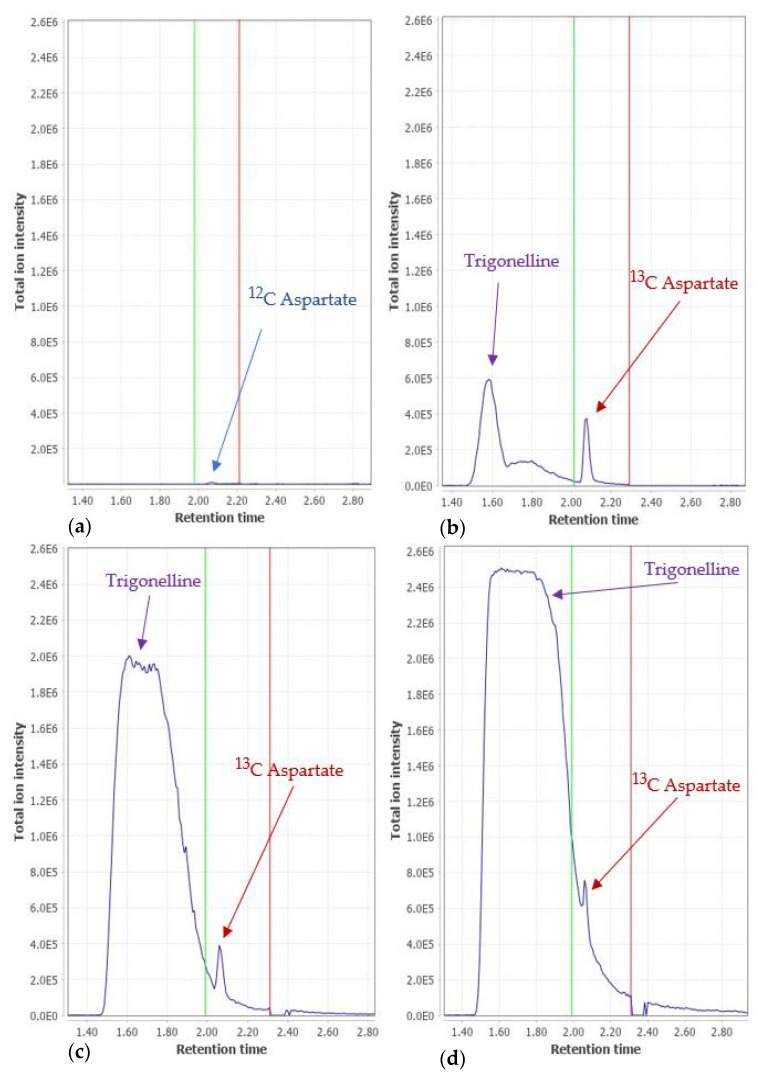
Extracted ion chromatogram for (**a**) ^12^C aspartate (*m*/*z* 134.0467 ± 0.003); (**b**) ^13^C aspartate (*m*/*z* 138.0578 ± 0.003) of a sample with a low trigonelline signal; and (**c**,**d**) ^13^C aspartate trace (*m*/*z* 138.0578 ± 0.003) for two samples with a high trigonelline signal. The green and the red vertical lines define the automatically integrated peak area.

A complex matrix such as urine that is further mixed with a complex labeled yeast extract poses a challenge for automated data analysis. One example is the case of aspartate. In both Figure 2 and Figure 4, aspartate shows a better correlation to quant without the IS. When inspecting the chromatographic peaks, we notice two main problems: there are samples where the ^12^C aspartate peak is so low in abundance that it falls within the noise, see Figure 6a. This explains the generally lower correlation even in “TOF absolute”. Furthermore, a peak with an *m/z* of 138.0549 elutes close to aspartate. The mass difference to ^13^C aspartate (*m/z* 138.0578) is below the 3 mDa tolerance used in data processing, hence resulting in an interference. The tentative identification of the neighboring peak suggests the ^12^C peak of trigonelline, a product of niacin (vitamin B3) metabolism, which is excreted in urine. The abundance of trigonelline varies across the samples. Figure 6b–d show that the larger the peak of trigonelline, the higher the interference with the ^13^C aspartate coming from the IS. While using a stricter *m/z* tolerance might solve this problem, it could cause problems in data processing for other compounds. This is in agreement with the work by Qiu et al. [34], who showed the advantage of higher resolution MS instrumentation for peak detection and identification.

Furthermore, employing a labeled cell extract as an internal standard will cause low abundant metabolites to have low signal intensities of the respective labeled compounds, resulting in a higher variance or difficulties in data integration.

**Figure 7:**
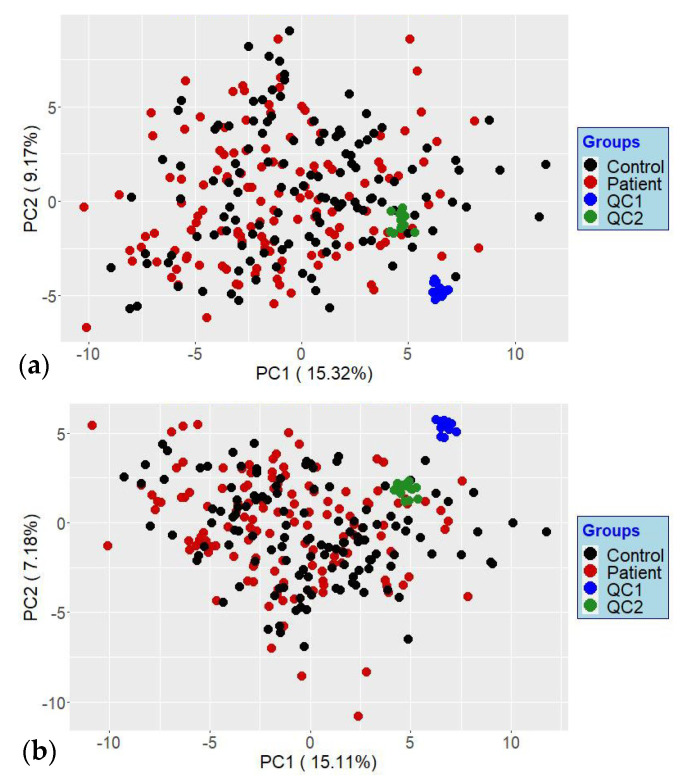
PCAs score plots of the analyzed sample set and the two QCs using all 112 metabolites, using absolute peak areas (**a**) and then the ratios to respective ISs (**b**). Notice that the QCs injections (14 each) cluster in both cases together. Note that the analyzed sample set corresponds to two groups of GCKD patients termed control and patients.

In general, using a yeast extract for urine analysis is not optimal. We, however, did not address the other aspects of using the kit here, such as the range of covered metabolites, which would be readily affected by the matrix type. We, instead, focus on a few identified metabolites and investigate the use of a complex IS and its ability to improve the data correlation to a reference dataset. The lack of improvement when using ratios instead of absolute peak areas can also be explained by an analysis of stability. We did not observe any shifts or the deterioration of the HPLC–TOFMS performance when analyzing the 244 samples. This is exemplified by the PCA scores plot shown in Figure 7a. The QC samples, two urine samples analyzed 14 times each, cluster closely together.

Regarding the batch effects, the application of the kit helped to reduce them. Nevertheless, the IROA–ratio list might show even better results when comparing several batches measured over a prolonged time or even measured on different instruments. Additionally, since RBE works when there are distinct batches, IROA ratios would be more beneficial if samples are measured in a single batch over a long period of time and a gradual decline in instrument performance occurs throughout the batch. This, however, is beyond the scope of this study.

## 5. Conclusions

Using a complex ^13^C labeled yeast extract enabled the analysis of 112 metabolites using the ratio approach. While the correlation with reference data did not improve significantly, the ratio approach helped to reduce the batch effects. However, most features in the data set are not covered by the approach due to a missing internal standard signal, which is also due to the mismatch between the sample (urine) and internal standard matrix (yeast). Moreover, complications arising from simultaneously handling a vast number of features in the untargeted approach undermine the benefits of incorporating this IS into our workflow. Matrix interference; co-eluting isobaric compounds; and imprecisions in peak integration, -alignment, and -gap–filling are all potential factors. Their impact can be mitigated by means of improved chromatography, cutting-edge MS instrumentation, upgraded processing software, and tailored post processing algorithms.

## Figures and Tables

**Table 1:** Dilution of urine samples based on creatinine concentration.

Creatinine (mM)	Dilution Factor
1–3	undiluted
3–6	1:2
6–12	1:4
12–24	1:8

**Table 2:** Spearman correlation coefficients for TOF absolute vs. quant and TOF ratios vs. quant.

Metabolite	TOF Absolute	TOF Ratios
arginine	0.694	0.8224
aspartate	0.505	0.407
glutamate	0.917	0.9118
glutamine	0.919	0.879
lysine	0.761	0.843
phenylalanine	0.978	0.9894
proline	0.559	0.5985
serine	0.858	0.8811
threonine	0.890	0.8044
tryptophan	0.922	0.9613
tyrosine	0.980	0.9782

**Table 3:** Paired Wilcoxon or *t*-test results comparing “diff” values between TOF absolute and TOF ratios against quant.

Metabolite	*p*-Value	Paired Test Type
arginine	0.735	Wilcoxon
aspartate	0.999	*t*-test
glutamate	1.000	*t*-test
glutamine	0.971	Wilcoxon
lysine	0.999	*t*-test
phenylalanine	0.997	Wilcoxon
proline	0.999	*t*-test
serine	1.000	*t*-test
threonine	0.998	*t*-test
tryptophan	0.977	Wilcoxon
tyrosine	0.810	Wilcoxon

**Table 4:** Spearman correlation coefficients for “TOF absolute” vs. “NMR” and “TOF ratios” vs. “NMR”.

Metabolite	TOF Absolute	TOF Ratios
aspartate	0.456	0.333
lysine	0.669	0.625
phenylalanine	0.720	0.767
threonine	0.789	0.673
tyrosine	0.849	0.849
tryptophan	0.733	0.775

**Table 5:** Paired Wilcoxon or *t*-test results comparing “diff” values between “TOF absolute” and “TOF ratios” against “NMR”.

Metabolite	*p*-Value	Paired Test Type
aspartate	0.999	*t*-test
lysine	0.999	*t*-test
phenylalanine	0.977	Wilcoxon
threonine	0.861	Wilcoxon
tyrosine	0.996	*t*-test
tryptophan	0.998	*t*-test

## Data Availability

The data presented in this study are available on request.

## Supplementary Materials

The following supporting information can be downloaded at: https://www.mdpi.com/article/10.3390/metabo12080741/s1. Table S1: Summary of detected metabolites across all three datasets; Table S2: Shapiro normality test results for TOF AAs data series of 56 samples each.; Figure S1: Spearman correlation plots of NMR vs. quant for the six overlapping AAs (Ranks are shown); Figure S2: Average concentration (µM) of the AAs in the subset of 56 samples, from “quant” data; Figure S3: Histograms of the relative standard deviations of the features’ peak areas averaged from all QC1 and QC2 injections; Table S3: Results of Shapiro test of normality for the relative standard deviation of the features in QCs; Table S4: Results of Wilcoxon test for the relative standard deviation of the features in QCs; Table S5: Results of Shapiro and Wilcoxon test for the relative standard deviation of the features in QCs; Figure S4: Extracted ion chromatograms of *m*/*z* 91.113, the IS of *m*/*z* 86.096, from two injections of QC2.

## References

[1] Doerr A. (2017). Global metabolomics. Nat. Methods.

[2] Fiehn O. (2002). Metabolomics—The link between genotypes and phenotypes. Plant Mol. Biol..

[3] Dudzik D., García A., Wood P.L. (2021). Untargeted Metabolomics Methods to Analyze Blood-Derived Samples. Metabolomics.

[4] Dettmer K., Hammock B.D. (2004). Metabolomics—A new exciting field within the "omics" sciences. Environ. Health Perspect..

[5] Dettmer K., Aronov P.A., Hammock B.D. (2007). Mass spectrometry-based metabolomics. Mass Spectrom. Rev..

[6] De Jong F.A., Beecher C. (2012). Addressing the current bottlenecks of metabolomics: Isotopic Ratio Outlier Analysis™, an isotopic-labeling technique for accurate biochemical profiling. Bioanalysis.

[7] Alseekh S., Aharoni A., Brotman Y., Contrepois K., D’Auria J., Ewald J., Ewald J.C., Fraser P.D., Giavalisco P., Hall R.D. (2021). Mass spectrometry-based metabolomics: A guide for annotation, quantification and best reporting practices. Nat. Methods.

[8] Uppal K., Walker D.I., Jones D.P. (2017). xMSannotator: An R Package for Network-Based Annotation of High-Resolution Metabolomics Data. Anal. Chem..

[9] Hermann G., Schwaiger M., Volejnik P., Koellensperger G. (2018). 13C-labelled yeast as internal standard for LC-MS/MS and LC high resolution MS based amino acid quantification in human plasma. J. Pharm. Biomed. Anal..

[10] Klein S., Heinzle E. (2012). Isotope labeling experiments in metabolomics and fluxomics. Wiley Interdiscip. Rev. Syst. Biol. Med..

[11] Guo K., Li L. (2009). Differential 12C-/13C-isotope dansylation labeling and fast liquid chromatography/mass spectrometry for absolute and relative quantification of the metabolome. Anal. Chem..

[12] Guo K., Li L. (2010). High-performance isotope labeling for profiling carboxylic acid-containing metabolites in biofluids by mass spectrometry. Anal. Chem..

[13] Hsu J.-L., Chen S.-H. (2016). Stable isotope dimethyl labelling for quantitative proteomics and beyond. Philos. Trans. A Math. Phys. Eng. Sci..

[14] Yuan W., Anderson K.W., Li S., Edwards J.L. (2012). Subsecond absolute quantitation of amine metabolites using isobaric tags for discovery of pathway activation in mammalian cells. Anal. Chem..

[15] Wu L., Mashego M.R., van Dam J.C., Proell A.M., Vinke J.L., Ras C., van Winden W.A., van Gulik W.M., Heijnen J.J. (2005). Quantitative analysis of the microbial metabolome by isotope dilution mass spectrometry using uniformly 13C-labeled cell extracts as internal standards. Anal. Biochem..

[16] Bueschl C., Kluger B., Lemmens M., Adam G., Wiesenberger G., Maschietto V., Marocco A., Strauss J., Bödi S., Thallinger G.G. (2014). A novel stable isotope labelling assisted workflow for improved untargeted LC-HRMS based metabolomics research. Metabolomics.

[17] Dethloff F., Bueschl C., Heumann H., Schuhmacher R., Turck C.W. (2018). Partially 13C-labeled mouse tissue as reference for LC-MS based untargeted metabolomics. Anal. Biochem..

[18] Beecher C., de Jong F.A., Raskind A. (2019). IROA Technology Primer ClusterFinder™ V3 Software User Manual.

[19] Goh W.W.B., Wang W., Wong L. (2017). Why Batch Effects Matter in Omics Data, and How to Avoid Them. Trends Biotechnol..

[20] R Core Team (2020). R: A Language and Environment for Statistical Computing.

[21] Gentleman R. (2005). Bioinformatics and Computational Biology Solutions Using R and Bioconductor.

[22] Liu Q., Walker D., Uppal K., Liu Z., Ma C., Tran V., Li S., Jones D.P., Yu T. (2020). Addressing the batch effect issue for LC/MS metabolomics data in data preprocessing. Sci. Rep..

[23] Stupp G.S., Clendinen C.S., Ajredini R., Szewc M.A., Garrett T., Menger R.F., Yost R.A., Beecher C., Edison A.S. (2013). Isotopic ratio outlier analysis global metabolomics of Caenorhabditis elegans. Anal. Chem..

[24] Qiu Y., Kurland I.J., Wood P.L. (2021). High Accurate Mass Gas Chromatography–Mass Spectrometry for Performing Isotopic Ratio Outlier Analysis: Applications for Nonannotated Metabolite Detection. Metabolomics.

[25] Titze S., Schmid M., Köttgen A., Busch M., Floege J., Wanner C., Kronenberg F., Eckardt K.-U., Prokosch H.-U., for the GCKD study investigators (2015). Disease burden and risk profile in referred patients with moderate chronic kidney disease: Composition of the German Chronic Kidney Disease (GCKD) cohort. Nephrol. Dial. Transplant..

[26] Vogl F.C., Mehrl S., Heizinger L., Schlecht I., Zacharias H.U., Ellmann L., Nürnberger N., Gronwald W., Leitzmann M.F., Rossert J. (2016). Evaluation of dilution and normalization strategies to correct for urinary output in HPLC-HRTOFMS metabolomics. Anal. Bioanal. Chem..

[27] Van der Goot A.T., Zhu W., Vázquez-Manrique R.P., Seinstra R.I., Dettmer K., Michels H., Farina F., Krijnen J., Melki R., Buijsman R.C. (2012). Delaying aging and the aging-associated decline in protein homeostasis by inhibition of tryptophan degradation. Proc. Natl. Acad. Sci. USA.

[28] Chambers M.C., Maclean B., Burke R., Amodei D., Ruderman D.L., Neumann S., Gatto L., Fischer B., Pratt B., Egertson J. (2012). A cross-platform toolkit for mass spectrometry and proteomics. Nat. Biotechnol..

[29] Pluskal T., Castillo S., Villar-Briones A., Oresic M. (2010). MZmine 2: Modular framework for processing, visualizing, and analyzing mass spectrometry-based molecular profile data. BMC Bioinform..

[30] Wilcoxon F., Kotz S., Johnson N.L. (1992). Individual Comparisons by Ranking Methods. Methodology and Distribution.

[31] Shapiro S.S. (1965). An Analysis of Variance Test for Normality (Complete Samples). Biometrika.

[32] Leek J.T., Scharpf R.B., Bravo H.C., Simcha D., Langmead B., Johnson W.E., Geman D., Baggerly K., Irizarry R.A. (2010). Tackling the widespread and critical impact of batch effects in high-throughput data. Nat. Rev. Genet..

[33] Dieterle F., Ross A., Schlotterbeck G., Senn H. (2006). Probabilistic quotient normalization as robust method to account for dilution of complex biological mixtures. Application in 1H NMR metabonomics. Anal. Chem..

[34] Qiu Y., Moir R.D., Willis I.M., Seethapathy S., Biniakewitz R.C., Kurland I.J. (2018). Enhanced Isotopic Ratio Outlier Analysis (IROA) Peak Detection and Identification with Ultra-High Resolution GC-Orbitrap/MS: Potential Application for Investigation of Model Organism Metabolomes. Metabolites.

