# Correction: Fadil et al. Isotope Ratio Outlier Analysis (IROA) for HPLC–TOFMS-Based Metabolomics of Human Urine. *Metabolites* 2022, *12*, 741

**DOI:** 10.3390/metabo14060293

**Published:** 2024-05-23

**Authors:** Fadi Fadil, Claudia Samol, Raffaela S. Berger, Fabian Kellermeier, Wolfram Gronwald, Peter J. Oefner, Katja Dettmer

**Affiliations:** Institute of Functional Genomics, University of Regensburg, 93053 Regensburg, Germany; fadi.fadil@ukr.de (F.F.); claudia1.bogner@ukr.de (C.S.); raffaela.berger@ukr.de (R.S.B.); fabian.kellermeier@ukr.de (F.K.); wolfram.gronwald@ukr.de (W.G.); peter.oefner@ukr.de (P.J.O.)

## Clarification

It was pointed out to us that we had not followed exactly the IROA TruQuant IQQ Workflow Kit protocol in the experimental part of our work [1]. In the paper, we do not indicate that we used the IROA TruQuant IQQ Workflow Kit protocol. We state that we purchased the “IROA TruQuant IQQ Workflow Kit” (which is the product name) through Sigma–Aldrich. Several different protocols have been published for the IROA TruQuant IQQ Workflow Kit [2,3,4] or employed in recent works [5,6,7].

Nevertheless, we would like to emphasize that we had deviated from the protocol of the IROA TruQuant IQQ Workflow Kit provided by the manufacturer for good reason, as we wanted to avoid drying of the urine specimens, which resulted in a lower IS concentration than recommended. A detailed comparison of the dilutions we used, and the IROA TruQuant IQQ Workflow Kit protocol given in the product information sheet [8], respectively, in the kit manual (022022IROAWORKFLOW) [9] is given in Table 1. Recently, the recommended protocol was changed (12172022IROAWORKFLOW) [10]. The newest parameters are also given in Table 1 for comparison.

**Table 1:** Comparison of protocol steps.

	Protocol Employed in Fadil et al. [1]	IROA TruQuant IQQ Workflow Kit Protocol before 12/2022 [8,9]	IROA TruQuant IQQ Workflow Kit Protocol after 12/2022 [10]
Volume to reconstitute LTRS/vial	80 µL	40 µL	30 µL
Volume to reconstitute IS/vial	600 µL	1200 µL	900 µL
Sample volume	20 µL	40 µL	30 µL
Sample dried	no	yes	yes
Volume of IS per sample	Urine sample is mixed with concentrated IS: 20 µL urine and 10 µL IS	40 µL to reconstitute dried sample	30 µL to reconstitute dried sample

In the interest of completeness, we performed additional experiments following the kit protocol [8,9]. We were also provided with an upgraded version of the proprietary software ClusterFinder™ V4.2.11A (CF4). The supplement is now updated with additional data acquired using the suggested protocol (022022IROAWORKFLOW). Furthermore, the data were analyzed using CF4 (parameters in Table S6). A calibration test was performed (Figure S5), based on which suited urine specimens were reanalyzed. The corresponding Spearman correlations are shown (Table S7). The ability to correct for batch effects was also retested (Figure S6).

An addition has been made to Materials and Methods, 2.2. Sample Preparation:

To investigate whether the urine specimens used in the experiment contained a sufficient concentration of metabolites, we performed the recommended calibration experiment according to the protocol in the Kit’s product information sheet [27]. The content of the internal standard (IS) vial was solubilized in 1.2 mL pure water and that of the LTRS in 40 μL pure water as suggested in the protocol. A urine specimen with a high creatinine level (20 mM) was diluted with pure water to creatinine concentrations of 0.25, 0.5, 1.25, 1.5, 2, 2.5, 3.75, 5, 7.5, 10, and 20 mM, respectively. Forty-microliter aliquots of each dilution were dried in triplicate employing an infrared vortex vacuum evaporator (CombiDancer, Hettich AG, Baech, Switzerland) and reconstituted in 40 μL IS solution.

As a result, a graph (see Figure S5) has been obtained that indicates the creatinine concentration to which urine specimens shall be adjusted. According to the product information sheet [27], this concentration “*yields an overall mass spectral signal that is equal to the overall mass spectral signal of the IS. This is the amount of sample that will most accurately be measured using the IS in the future, i.e., well balanced by the standard 40 μL of IS.*” In Figure S5, this is the intersection between the lines of the normalized IS MSTUS (blue squares), the normalized ^12^C MSTUS (green crosses), and the line of the ^12^C values corrected for ion suppression (red circles). In our case, this corresponds to about 6.5 mM creatinine. We then repeated the original experiment using a subset of 26 urine specimens with original creatinine concentrations equal to or greater than 6.5 mM. Aliquots of 40 μL of urine with a creatinine concentration of 6.5 mM (either pure or prediluted to 6.5 mM) were dried and reconstituted in 40 μL IS (dissolved according to protocol in 1.2 mL) so that each sample contained a final creatinine concentration of 6.5 mM. The LTRS and IS blanks were also prepared according to the protocol. Additionally, aliquots from the same 26 urine specimens were diluted to 2 mM creatinine to obtain concentrations comparable to our original data set, and aliquots of 40 μL were also dried and reconstituted in 40 μL IS.

An addition has been made to Materials and Methods, 2.5. Software:

We were provided with an upgraded version of the proprietary software ClusterFinder™ V4.2.11A (CF4). This was used for the analysis of the calibration experiment, the creatinine concentration investigation, and the analysis of the original samples that were employed for the investigation of batch effects. The parameters used are listed in Table S6. As the kit is based on 95% ^13^C for the IS, a noticeable m-1 peak (and m-2 depending on the C-number, etc.) is formed for the IS signal that increases with carbon number. For a semi-quantitative comparison of the endogenous metabolite amount to the IS amount in the sample, the peak areas of the isotopologue envelops are summed up in CF4. CF4 calculates the complete ratio between the sum of natural abundance isotopologues and the sum of 95% ^13^C isotopologues, in contrast to considering only the monoisotopic peak in each envelope of isotopologues when using MZmine. Since the absolute amount of substance of the metabolites in the IS is not known, we cannot compare the quantities of different metabolites in a sample to each other. In our work, we only compared the relative levels of the same metabolites between samples. As the isotope pattern does not change for a given metabolite in the IS or the urine specimen between samples, using the monoisotopic peak ratio is, in our opinion, a sufficient substitute for the summed-up isotopologue envelops. For comparison, the new datasets for the 26 urine specimens diluted either to 2.0 or 6.5 mM creatinine were analyzed with both CF4 and with MZmine.

An addition has been made to the Results:

The original and new LTRS measurements (from the creatinine concentration test) were both analyzed separately by CF4 with the same parameters. The new LTRS measurements yielded 417 complete bins (uncured), compared to 232 complete bins (uncured) for the original dataset (higher dilution of the LTRS).

In the results file from CF4, three types of relevant outputs are considered: Raw, i.e., the absolute ^12^C peak area, corresponds to “TOF absolute”; ^12^C/^13^C Ratios that correspond to “TOF ratios”; and Suppression Corrected, i.e., peak areas that are corrected for ion suppression, as the name states. To this end, in CF4, the ^12^C/^13^C ratio is multiplied by the corresponding least suppressed ^13^C area. Spearman correlation coefficients with results obtained by a targeted MS analysis (see Section 2 for details) were calculated for both 2 mM and 6.5 mM creatinine, employing all three outputs. The results are shown in Table S7. For comparison, the corresponding coefficients from analyzing the same samples in MZmine are given.

The ability to correct for batch effects was considered again by reanalyzing the same original two batch effect datasets using CF4. Four relevant outputs were considered, i.e., Raw, Ratios, Suppression Corrected, and Normalized (MSTUS). The resulting PCAs are shown in Figure S6.

The newly added Supplementary Materials and reference appear below:

**Table S6:** Parameters used in CF4 (V4.2.11A) to build the LTRS library (discovery phase) and to conduct the semi-targeted search.

Discovery Phase		Semi-Targeted IROA Search	
M/Z tolerance, ppm	20	M/Z tolerance, ppm	30
Limit retention time range, min	0.5–18	Limit retention time range, min	0.5–12
Smoothing filter width	9	Smoothing filter width	9
Minimum base peak intensity	5000	Minimum base peak intensity	10000
Exclude peaks below intensity	2500	Exclude peaks below intensity	5000
Signal to noise cutoff	3	Signal to noise cutoff	5
Peak shape correlation cutoff	0.3	Peak shape correlation cutoff	0.5
Minimal score for isotope pattern quality	30	Minimal score for isotope pattern quality	30
Precursor isolation window, Da	0.2	Precursor isolation window, Da	0.2
MSMS precursor M/Z tolerance, ppm	50	MSMS precursor M/Z tolerance, ppm	50
Relative intensity cutoff for MSMS lookup (0–1)	0.3	Relative intensity cutoff for MSMS lookup (0–1)	0.3
%^13^C incorporation in sample	95	%^13^C incorporation in sample	95
%^13^C incorporation in control	5	%^13^C incorporation in Control	1.1
Maximal charge to report	2	Require at least two ^12^C isotopes	no
Max. shift between ^12^C and ^13^C envelopes, min	0.1	Require at least two ^13^C isotopes	no
RT window for peak binning, min	0.2	RT window for peak binning	0.25
Peak width range, min	0.05–1	Max error in relative IROA isotopes intensity	30%
Smoothing window multiplier	1.5	Calculate complete isotopic pattern score	no
Create orphaned clusters	no	Try to identify unkowns	no
Calculate complete isotopic pattern score	no	Keep compound IDs from source for unkown compounds	yes
		Run reintegration	yes

**Table S7:** Spearman correlation coefficients for Raw, Ratio, Suppression corrected vs the quant data set according to the urinary creatinine concentrations chosen (subset of 26 samples). For comparison, the data were also analyzed using MZmine and the correlations are given. Shown are the mutual metabolites for the output of CF4 and MZmine.

Creatinine [mM]	6.5 mM CF4			2 mM CF4			6.5 mM MZmine		2 mM MZmine	
Method Used	Raw	Ratios	Suppression Corrected	Raw	Ratios	Suppression Corrected	Raw	Ratios	Raw	Ratios
**arginine**	0.43	0.88	0.88	0.48	0.67	0.67	0.66	0.55	0.43	0.73
**asparagine**	0.55	0.62	0.62	0.45	0.61	0.61	0.55	0.54	0.58	0.65
**aspartate**	0.57	0.13	0.13	0.53	0.11	0.11	0.49	0.10	0.57	0.12
**glutamine**	0.71	0.60	0.60	0.75	0.69	0.69	0.70	0.60	0.77	0.73
**histidine**	0.74	0.84	0.84	0.79	0.82	0.82	0.79	0.82	0.67	0.61
**lysine**	0.85	0.95	0.95	0.78	0.95	0.95	0.79	0.88	0.73	0.87
**methionine**	0.79	0.80	0.80	0.81	0.79	0.79	0.77	0.78	0.79	0.82
**phenylalanine**	0.89	0.92	0.92	0.91	0.90	0.90	0.89	0.90	0.91	0.89
**serine**	0.52	0.70	0.70	0.60	0.71	0.71	0.53	0.71	0.59	0.70
**threonine**	0.84	0.52	0.52	0.87	0.75	0.75	0.85	0.46	0.88	0.74
**tryptophan**	0.88	0.93	0.93	0.95	0.93	0.93	0.90	0.95	0.95	0.93
**tyrosine**	0.95	0.93	0.93	0.94	0.96	0.96	0.96	0.93	0.94	0.96

**Figure S5:**
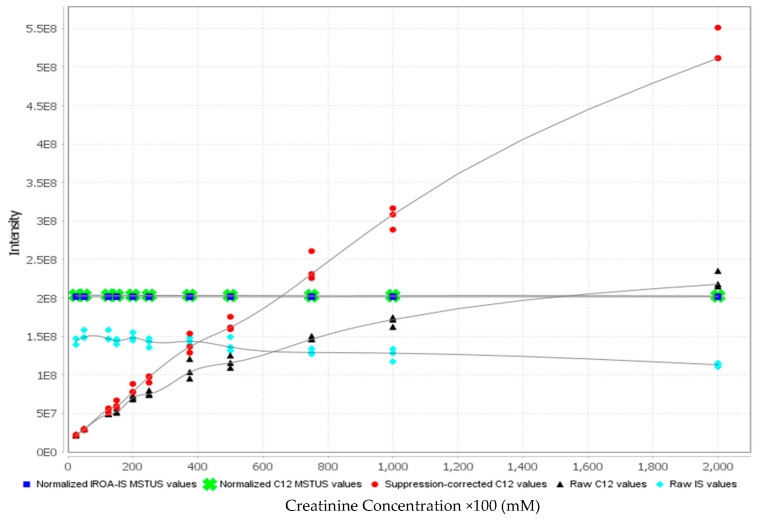
Calibration experiment results. Note that the X axis shows the creatinine concentrations in mM multiplied by a factor of 100. The graph was generated by CF4 (V4.2.11A).

**Figure S6:**
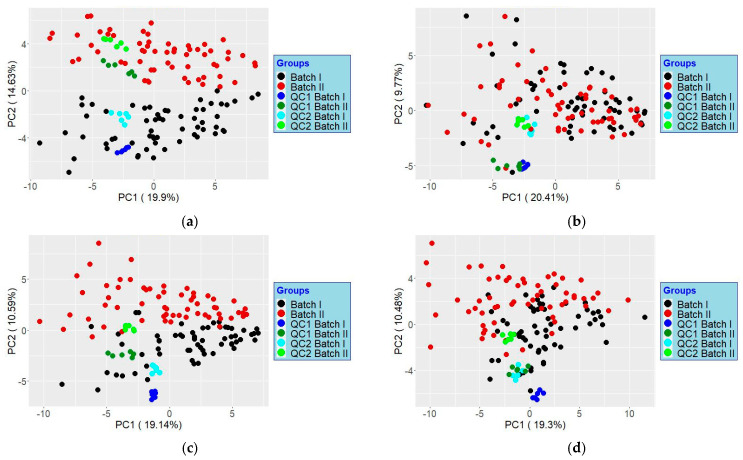
PCA score plots based on data analyzed using ClusterFinder™ V4.2.11A. (**a**) raw peak areas, (**b**) ^12^C/^13^C ratios; (**c**) suppression corrected; (**d**) normalized data. The tighter the scores cluster for a given QC from two batches, the better batch effects have been mitigated.

27. Sigma-Aldrich Co., LLC. Product Information, IROA TruQuant IQQ Workflow Kit. Supplied by IROA Technologies, LLC.: Catalog Number WORKFLOW. 2019. Available online: https://www.sigmaaldrich.com/deepweb/assets/sigmaaldrich/product/documents/362/924/workflowpis.pdf (accessed on 14 November 2019).

The corrected Supplementary Materials part is provided below:

**Supplementary Materials:** The following supporting information can be downloaded at: https://www.mdpi.com/article/10.3390/metabo12080741/s1. Table S1: Summary of detected metabolites across all three datasets; Table S2: Shapiro normality test results for TOF AAs data series of 56 samples each. Figure S1: Spearman correlation plots of NMR vs. quant for the six overlapping AAs (Ranks are shown); Figure S2: Average concentration (μM) of the AAs in the subset of 56 samples, from “quant” data; Figure S3: Histograms of the relative standard deviations of the features’ peak areas averaged from all QC1 and QC2 injections; Table S3: Results of Shapiro test of normality for the relative standard deviation of the features in QCs; Table S4: Results of Wilcoxon test for the relative standard deviation of the features in QCs; Table S5: Results of Shapiro and Wilcoxon test for the relative standard deviation of the features in QCs; Figure S4: Extracted ion chromatograms of *m*/*z* 91.113, the IS of *m*/*z* 86.096, from two injections of QC2. Table S6: Parameters used in CF4 to build the LTRS library (discovery phase) and to conduct the semi-targeted search; Table S7: Spearman correlation coefficients for Raw, Ratio, Suppression corrected vs. the quant data set according to the urinary creatinine concentrations chosen (subset of 26 samples); Figure S5: Calibration experiment results; Figure S6: PCA score plots based on data analyzed using ClusterFinder™ V4.2.11A.

With this correction, the order of some references has been adjusted accordingly. The authors state that the additional experiments performed have not changed the scientific conclusions of the original publication. This correction was approved by the Academic Editor. The original publication has also been updated.
