# Supplementary material for: Isotope Ratio Outlier Analysis (IROA) for HPLC–TOFMS-Based Metabolomics of Human Urine
Source: Metabolites. 2022 Aug 12;12(8):741. doi: 10.3390/metabo12080741 (PMC9414531; doi:10.3390/metabo12080741)
Supplement: Supplementary file 1 [file metabolites-12-00741-s001.zip › metabolites-1842870-supplementary.pdf]

Supplemental material

Table S1. Summary of detected metabolites across all three datasets. TOF unlabeled represents metabolites detected regardless of their fully labeled signal. TOF IROA IS represent the labeled IS of the corresponding metabolite, originating from the added IS of the kit.

Metabolite	m/z (M+H)	RT	in-house library	Quant	NMR
aspartate	134.047	2.06	Y	Y	Y
lysine	147.113	1.06	Y	Y	Y
phenylalanine	166.086	5.17	Y	Y	Y
threonine	120.065	1.39	Y	Y	Y
tryptophan	205.097	6.41	Y	Y	Y
tyrosine	182.081	4.03	Y	Y	Y
arginine	175.116	1.00	Y	Y	N
glutamate	148.061	1.9	Y	Y	N
glutamine	147.076	1.30	Y	Y	N
proline	116.070	1.40	Y	Y	N
serine	106.050	1.35	Y	Y	N
leucine	132.102	3.17	Y	Y	N
Isoleucine	132.102	2.97	Y	Y	N
acetyllysine	189.123	1.32	Y	N	N
adensosine	268.104	4.60	Y	N	N
adensosine 5'-monophosphate	348.071	5.49	Y	N	N
alpha glycerylphosphorylcholine	258.112	1.33	Y	N	N
alpha-aminoadipate	162.076	1.99	Y	N	N
choline phosphate	184.072	1.38	Y	N	N
homocysteine thiolactone	235.057	0.99	Y	N	N
methyl-aspartate	148.061	1.93	Y	N	N
methyl-glutamate	162.076	2.06	Y	N	N
methylthioadenosine	298.096	6.63	Y	N	N
mevalonolactone	131.070	4.90	Y	N	N
oxo-proline	130.050	5.33	Y	N	N
palatinose	325.112	1.70	Y	N	N
psicose	163.060	1.71	Y	N	N

Legend
Detected
Excluded
Not investigated

Table S2 Shapiro normality test results for TOF AAs data series of 56 samples each.

Data Series	Shapiro test p–value	statistical distribution	Suitable test
arginine Absolute	0.0297	non–normal	Wilcoxon
arginine Ratio	0.2627	normal	
aspartate Absolute	0.1612	normal	t–test
aspartate Ratio	0.5507	normal	
glutamate Absolute	0.1503	normal	t–test
glutamate Ratio	0.0920	normal	
glutamine Absolute	0.3569	normal	Wilcoxon
glutamine Ratio	0.0278	non–normal	
lysine Absolute	0.8874	normal	t–test
lysine Ratio	0.1137	normal	
phenylalanine Absolute	0.0076	non–normal	Wilcoxon
phenylalanine Ratio	0.0017	non–normal	
proline Absolute	0.5469	normal	t–test
proline Ratio	0.5168	normal	
serine Absolute	0.2816	normal	t–test
serine Ratio	0.0758	normal	
threonine Absolute	0.7258	normal	t–test
threonine Ratio	0.3951	normal	
tryptophan Absolute	0.0000	non–normal	Wilcoxon
tryptophan Ratio	0.0000	non–normal	
tyrosine Absolute	0.0005	non–normal	Wilcoxon
tyrosine Ratio	0.0014	non–normal	

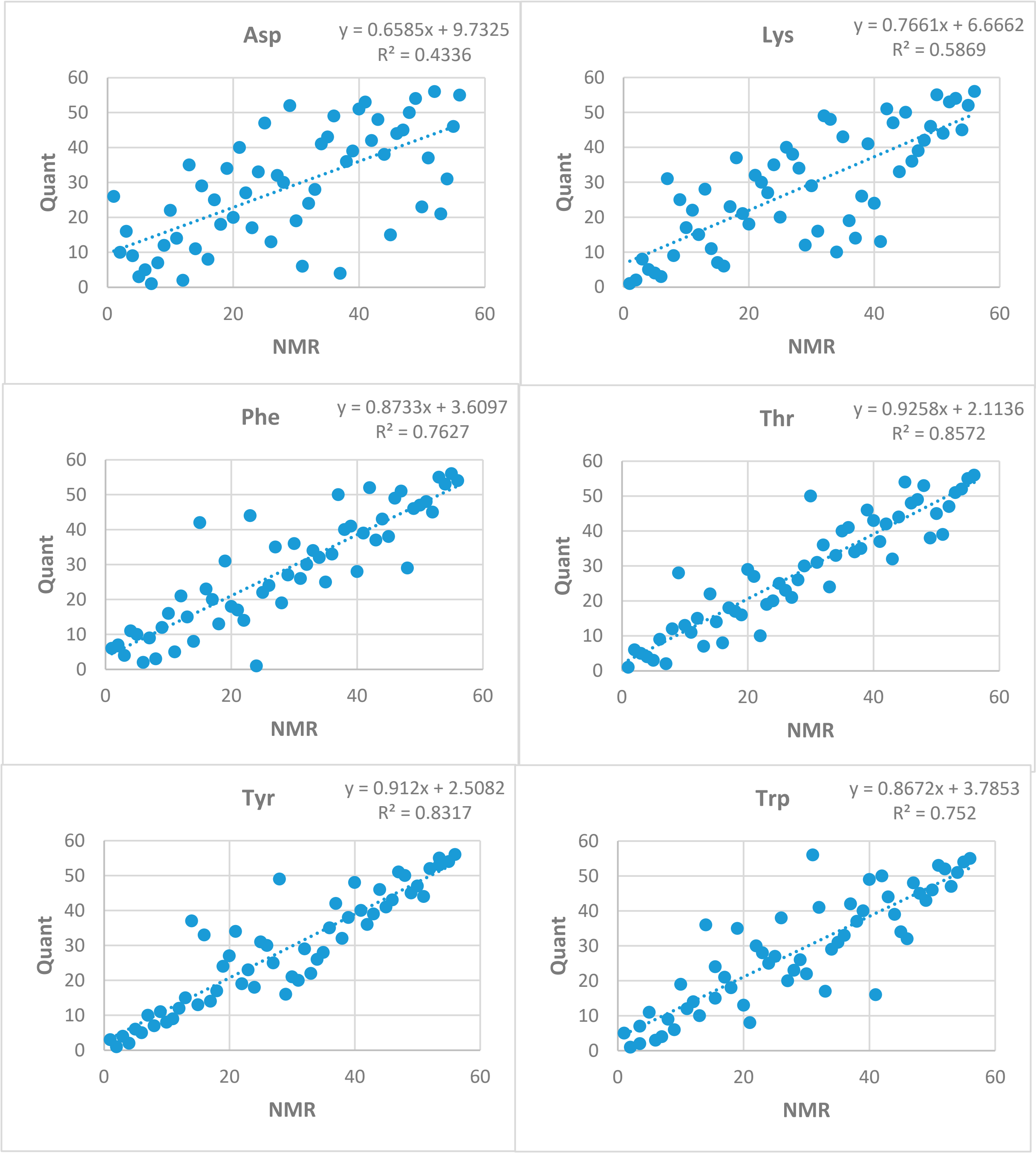

Figure S1 Spearman correlation plots of NMR vs. Quant for the six overlapping AAs (Ranks are shown).

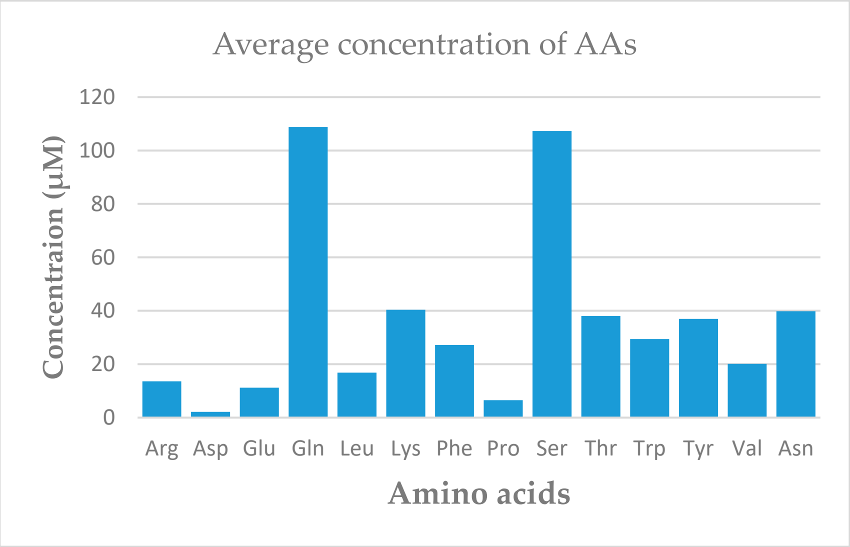

Figure S2 Average concentration (µM) of the AAs in the subset of 56 samples, from "Quant" data. Aspartate and proline show the lowest concentrations.

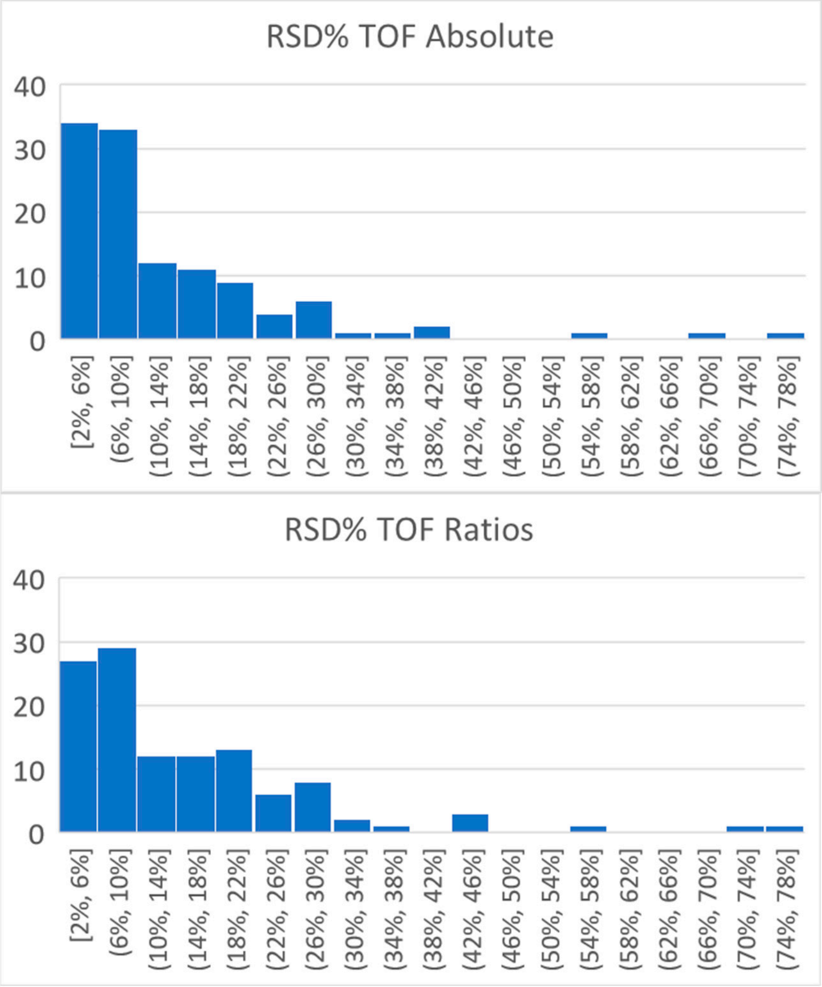

Figure S3. Histograms of the relative standard deviations of the features' peak areas averaged from all QC1 and QC2 injections; as absolute peak areas (top), and as ratios to the respective IS (bottom).

Table S3. Results of Shapiro test of normality for the relative standard deviation of the features in QCs.

RSD of the Dataset:	p-value Shapiro test
QC1_TOF_Absolute	6.90E-14
QC1_TOF Ratios	1.45E-11
QC2_TOF_Absolute	1.90E-15
QC2_TOF Ratios	4.51E-14
Average QC1+QC2_TOF Absolute	1.08E-13
Average QC1+QC2_TOF Ratios	6.56E-12

Table S4. Results of Wilcoxon test for the relative standard deviation of the features in QCs.

RSD TOF Absolute vs. TOF Ratios	p-value paired Wilcoxon test
Average QC1+QC2	2.67E-05
QC1	2.63E-04
QC2	2.37E-04

Table S5. Results of Shapiro and Wilcoxon test for the relative standard deviation of the features in QCs. The features were divided into quantiles based on the peak area of the IS, with Q1 having the highest and Q4 the lowest peak areas. Notice the

	Q1	Q2	Q3	Q4
p-value Shapiro test TOF_Absolute	1.05E-05	5.00E-03	1.65E-06	8.85E-06
p-value Shapiro test TOF_Ratios	5.00E-04	4.70E-03	1.21E-05	1.47E-04
p-value paired Wilcoxon test TOF_Absolute vs TOF Ratios	9.15E-01	1.21E-02	2.15E-03	4.11E-03

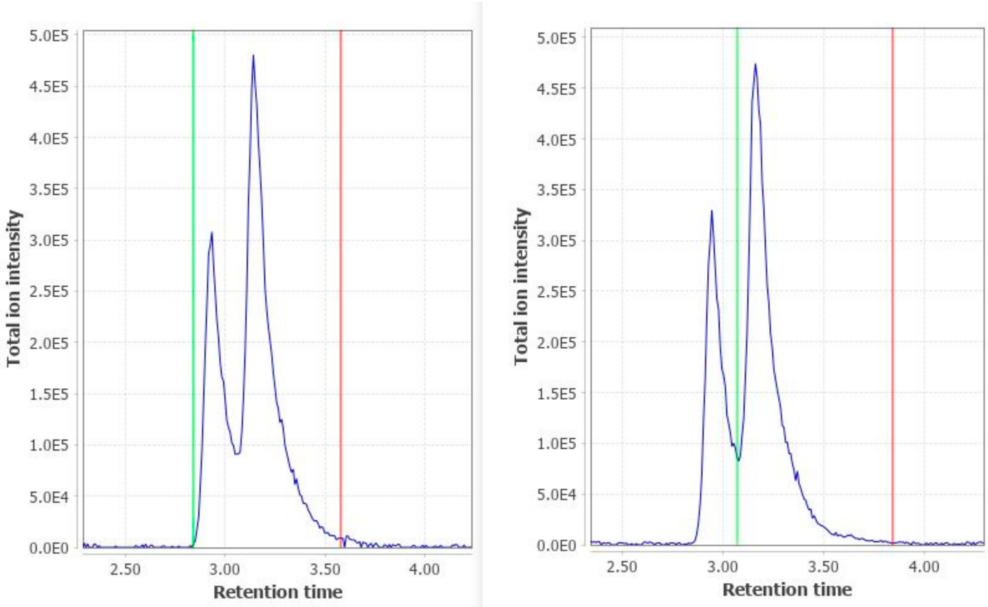

Figure S4. Extracted ion chromatograms of m/z 91.113, the IS of m/z 86.096, from two injections of QC2. The green and red automatic integration lines define different peaks due to the interference of the closely eluting isomer.
